# Needs and Expectations of Long-Term Cancer Survivors: Multi-Centre Study Protocol

**DOI:** 10.3390/jpm14010064

**Published:** 2024-01-04

**Authors:** Antonio Zamudio-Sánchez, Pilar Camarero-Gómez, Estefanía Rodríguez-Manjón, María Rosa Iglesias-Parra, Juan Gómez-Salgado, Adolfo Romero

**Affiliations:** 1 Internal Medicine Clinical Management Unit, Hospital Regional Universitario of Malaga, Instituto de Investigación Biomédica de Málaga (IBIMA), University of Malaga, 29010 Malaga, Spain; antonio.zamudio.sspa@juntadeandalucia.es (A.Z.-S.);; 2 Malaga Guadalhorce Health District, Instituto de Investigación Biomédica de Málaga (IBIMA), 29010 Malaga, Spain; 3 Department of Nursing and Podiatry, Instituto de Investigación Biomédica de Málaga (IBIMA), University of Malaga, 29010 Malaga, Spainadolforomeror@uma.es (A.R.); 4 Department of Sociology, Social Work, and Public Health, Faculty of Labour Sciences, University of Huelva, 21007 Huelva, Spain; 5 Safety and Health Posgrade Program, Espíritu Santo University, Samborondón 09-01-952, Ecuador

**Keywords:** cancer survivors, unmet needs, study protocol, patient-reported outcome measures, health-related quality of life, disease surveillance, epidemiology

## Abstract

Cancer is a social issue as its outreach affects not only mortality (it is the second cause of death in our environment) but also the costs due to morbidity and the distress it causes, as well as the losses and consequences in personal, family, work, and even social areas. This study is trying to find out the health needs of long-term cancer survivors and their perceptions and expectations of the care they received during their survival stage. For this, a joint, cross-sectional descriptive study with a qualitative and quantitative approach has been designed. For the qualitative approach, we have used different focus groups representing different geographical areas of the Spanish territory. For the qualitative approach, we have used a validated questionnaire. This study will provide a better knowledge of the quality of life of these patients, as well as their level of unmet and even unexpressed needs, in order to develop effective strategies and interventions that allow for the implementation of adapted care plans that include such unexpressed needs. This study will also allow for the creation and development of assessment methods for health results from the patient’s perspective and experience. These issues require a multidisciplinary, complex approach. These survivors may require not well-known health services, as the number of these patients has grown recently, and their survival time is also longer. This research explores a wider and more thorough perspective of long-term cancer survivors’ needs, experiences, and expectations to be achieved.

## 1. Introduction

Cancer is an obvious global health problem. It is one of the main causes of morbi-mortality worldwide, with approximately 14 million new cases in the world in the year 2012 [1].

Cancer is also considered a social disease, as its outreach affects not only mortality (it is the second cause of death in our environment) but also the costs due to the morbidity and distress it causes, as well as the losses and consequences in the personal, family, work, and even social areas. These issues require a multidisciplinary, complex approach [2,3].

In terms of individual risk, 1 out of 3 Spanish men and 1 out of 5 Spanish women may develop cancer at some stage of their lives, although cancer reaches all members of our society, by directly suffering or having suffered the disease or by having affected family members or friends [4,5].

More cancer patients get cured every year and luckily there is a higher number of long-term survivors (cured patients who do not receive treatment and whose diagnoses were made at least five years ago) who require special monitoring coordinated with other specializations [3]. For this reason, we can confirm without doubt that the cancer survivor population is growing. This is thanks to many factors such as early diagnosis, medical and pharmaceutical advances, and better access to health services [2].

As for the current state of Spain in the number of survivors, the 5-year survival rate for men for all types of cancer except for non-melanoma skin cancer is 41.2%, whereas the relative survival rate, namely removing the effect of other causes of death, is 49.0%. For women, the rates are higher; the survival rate is 54.0% and the relative survival rate is 59.4% [5,6].

In the U.S.A., out of 13.7 million cancer survivors, it is estimated that more than 60% survived longer than 5 years, 40% longer than 10 years, and 15% longer than 20 years after diagnosis [7].

In Europe, there are statistical data comparable to the United States, in particular those from the EUROCARE (European Cancer Registry Study on Survival and Care of Cancer Patients) database, which controls 82 European registries from 23 countries. From 1978 to 2007, the overall survival rate was 52% (58% for women and 46% for men) [8].

As for Spain, the cancer approach has changed significantly over the last 25 years, achieving long survival thanks to the improvement of treatments and care, including relapse and second tumor monitoring and symptom and psychosocial needs control [2]. In this sense, it is vital to understand how patients can live with this process.

It is precisely during the long-term survival stage that more information regarding the needs, problems, and challenges of both patients and their relatives is needed, as well as more coordination and organization of health entities [3]. Scientific evidence reveals a substantial diversity of the models of care, with unclear results. More specific research on the care of cancer survivors is needed in order to better understand the advantages and disadvantages of the possible models of care [9,10].

Among the most prevailing needs are energy loss, fear of a possible relapse, and feelings of distress, anxiety, and concerns regarding the future. In addition, the majority of the participants of the CESC questionnaire for the assessment of unmet needs of cancer survivors stated they encountered problems regarding their work reinsertion, especially those under 40 years of age. They also stated that none of them received assistance in order to solve these issues [11,12].

A report published by the Institute of Medicine of the United States (IOM), known as “From Cancer Patient to Cancer Survivor: Lost in Transition” [13], addressed this issue and exposed the lack of attention and research regarding this population group. In support of the IOM’s initiative, The American Center for Disease Control and Prevention (CDC) and the Lance Armstrong Foundation, renowned for its outstanding work in supporting cancer patients and conducting research during the survival stage, recommended the implementation of a specific care plan for cancer survivorship [14]. As for Spain, there has also been a proposal for the development and implementation of a plan of care for survivors that includes information about the type of cancer, stage, and therapies implemented, as well as complications, sequence, and content of the follow-up, all provided through multidisciplinary and interdisciplinary teams that promote a special, coordinated follow-up [3,15,16]. All of this requires an improvement of the strategies for the approach to this issue, including more and better research in our field, with regard to what other countries have done. An example of this is the study by Rowland et al., which compared the evolution of survivorship research in the U.S.A. and Europe [15].

Over the last years, there has been an attempt to incorporate the so-called “patient experience” in assistance processes in order to improve them, adapting them as much as possible to the life circumstances of people. The patient-reported outcome (PRO) is a general term that includes a series of final measure items in order to describe the results collected directly from the patient, which do not allow for the interpretation of the healthcare team [17,18]. For the assessment of these results, patient-reported outcome measures (PROMs) are used. PROMs are tools that measure the patient’s health state as the quality of life related to health. These tools are usually questionnaires filled out by the patient [19].

The patient experience plays a key role in clinical research conducting a comprehensive assessment of the impact of health care [20]. PROMs have become tools that guarantee a valid, reliable measure of these patient-reported outcomes. The inclusion of PROMs in clinical results of clinical research and practice provides a more thorough understanding of the impact that a particular intervention, treatment, or care has on a patient. This measure should present a positive association with clinical effectiveness and patient safety. A dissociation between them is not recommended [19].

On the other hand, patient-related experience measures (PREM) are tools that provide information about the level of satisfaction of the patient with a health system, and they are generic tools that are often used to apprehend the general patient experience with health care [21]. These tools have revealed positive links between patient satisfaction and clinical safety and are a reliable measure for the assessment of the quality level of the assistance in a health centre from the patient’s perspective [19,22,23]. Internationally, PREMs have been used to assess health care in terms of clinical effectiveness and economic efficiency [21]. In this sense, there is a growing international focus regarding the use of PREMs as an indicator of the quality of the attention and safety of the patient [24].

On the other hand, we must state that within the health field, there is growing interest in qualitative methodology, and its development and incorporation have been improved as a method of study and research. Nonetheless, we must point out that the implementation of qualitative findings differs from that of quantitative findings, as they do not always provide solutions to immediate practical problems, but can occasionally allow for a greater and better understanding of the environment and experience of the patient. Such understanding has, in turn, a practical effect: it allows for access to an action, that, in the words of Strauss, is moral and effective, as it will be based on the knowledge of the true nature of multiple, people-interpreted reality [14,25].

These backgrounds pose as a research purpose the increase of knowledge regarding health needs perceived by long-term cancer survivors and their expectations regarding the attention received during their survivorship stage.

### Specific Aims

- Knowing health results from the long-term cancer survivor perspective.
- Studying health results from long-term cancer survivor measurements.
- Assessing the perceived health needs and expectations of long-term cancer survivors depending on the type of cancer.
- Analysing the possible differences in needs and expectations according to place of origin, residence, affiliation with patients’ associations, time of survivorship, and health care type (specialist care and primary health care).
- Knowing the survivors’ requirements regarding health care services (specialist and primary health care).
- Stating potential user profiles depending on their care expectations, geographical areas, and socio-demographic data.
- Setting effective strategies that allow us to implement health results from the users’ perspective and experience.

## 2. Materials and Methods

### 2.1. Design

We designed a mixed study by means of both a quantitative and qualitative approach. The qualitative step will be performed by the setting of focal groups as PREMs tools; and the quantitative approach by using the CESC questionnaire regarding the needs of people who have completed their cancer treatment [11] as PROMs and PREMs tools in order to assess the patient health state from their perspective and experience, as well as their level of satisfaction and care quality.

This study will last 18 months and will be developed sequentially, with a first phase of gathering and analysis of quantitative data, which will be added to the qualitative part. This way, a wider and more thorough perspective of the long-term cancer survivors’ needs, experiences, and expectations will be achieved. In addition, the joint design will provide more soundness to the scientific inferences compared to studies with only one type of analysis [26].

### 2.2. Scope of Study

Multi-centre, nationwide level study with the involvement of 10 Spanish hospitals (Table 1). All health centres are part of the Spanish public health system, which is the reference system for cancer treatment all across Spain. All of these hospitals have more than 400 hospital beds and 80% of them have more than 700, favouring a diversity of circumstances, including cultural, economic, and health circumstances, as it covers all of the Spanish territory. The data collection will take place in medical cancer units.

### 2.3. Study Population

The study will include long-term cancer survivors treated in the 10 participating hospitals. As a clarification, the term ‘long-term cancer survivors’ includes those patients who were diagnosed with cancer more than five years prior to the study. In any case, the interested parties are health institutions in general (both hospital care and primary care) and patients, since in both cases the aim is to improve the care provided, optimizing the resources available for the care of long-term cancer survivors.

### 2.4. Sampling

The sample selection will depend on the phase of the study. However, the inclusion and exclusion criteria (Table 2) will be the same in both stages.

### 2.5. First Study Phase

During the first study phase (quantitative), patients monitored from the cancer areas of the hospitals involved will be chosen.

Depending on the number of cases attended and the patient survivorship, the sample required will be estimated in order to achieve the statistical significance of the data. The survivorship rate after 5 years refers to the percentage of patients who live at least 5 years after the cancer diagnosis. According to this background and the cancer incidence during that period of time, representative groups will be chosen in each hospital participating in the study, conducting a random stratified sampling. This way, with a confidence level of 95%, an alpha error of 0.5%, and an estimated loss percentage of 20%, we foresee the inclusion of a total of 570 patients.

A uniform stratified sampling will be conducted in every hospital, so that each one will choose up to 57 individuals, half of whom are still under hospital care and the other half are exclusively monitored by the primary health care system. This will allow for a more precise analysis of the particular features of each hospital and health area. In order not to overstate the presence of patients from a particular region, a consideration depending on its reference population will be conducted. Patient recruitment will be conducted after an evaluation of their clinical records in the Information Systems of each hospital, in order to confirm the inclusion criteria and contact them by telephone. This first study phase will be carried out for 7 months, from February 2024 to August 2024.

#### 2.5.1. Data Collection of the First Study Phase

The CESC questionnaire for the needs of people who have completed cancer treatment will be used, validated in the Spanish language [11]. The questionnaire contains 38 questions which, apart from assessing sociodemographic data and the care received, explore the physical, emotional, social, and economic dimensions of the patients. The CESC questionnaire, one of the few questionnaires developed in Spanish, meets the methodological standards regarding validity and reliability required to become an instrument in its first version. Other studies [27] have adapted and validated the tools for the assessment of cancer patient’s needs, where experience and needs differ from those patients in the treatment or palliative care stages, such as QLACS—a scale that measures the quality of life of cancer survivors [28] and CaSUN—a tool that assesses cancer survivors’ unmet needs [29]. These studies were developed with patients suffering from different types of cancer, with a sample of 34 people. From the Cancer Survivor’s Unmet Needs questionnaire (CaSUN-S), a validation of their psychometric properties was conducted [30] with a greater sample, but only in breast cancer survivors. Due to the fact that the CESC is a self-administered, easy-to-complete instrument, it can be incorporated into clinical practice for the identification of the unmet needs of these patients or needs that may change over time. The population recruited for the development of the CESC questionnaire was patients from different parts of Spain belonging to the Spanish Organization of Cancer Patients (GEPAC) who were diagnosed with a cancer disease and who completed the relevant treatment at least 1 year before taking part in the study. They also completed another questionnaire for the collection of PRO and PRE. The patients included in this study will be summoned for the completion of the questionnaire in a personal interview conducted in the participating hospitals. The researcher from each hospital will be responsible for informing, answering questions, and obtaining informed consent from the patients. Patients will be allowed to complete the questionnaire at the hospital or by phone call. The data will be kept by the researchers of each hospital and made anonymous, as stated by the law.

#### 2.5.2. Data Analysis of the First Study Phase

As a first step, a descriptive data analysis will be conducted, assessing measures of central tendency and dispersion (averages, standard deviations in a normal distribution, medians and quartiles in a non-normal distribution). Categorical variables will be presented as frequencies and percentages (Table 3).

In order to assess the normal or non-normal distribution of the data, the Kolmogorov–Smirnoff test will be conducted.

In order to analyse if the differences observed in the frequencies of the variables of interest are statistically significant, the Chi-square test will be conducted for qualitative variables, or Fisher’s exact test will be used when the percentage of expected values under 5 is above 20%.

In order to determine the results of the goal, the potential users’ profiles depending on their care expectations, geographical areas, and socio-demographic data will be collected and cluster analysis will be applied, using the Ward method to obtain hierarchical clusters. This technique will permit the establishment of case groups or relatively homogeneous observations. The Ward method starts by assuming every data point forms a group, and it joins the elements by the minimum increase of W, which implies taking the closest elements to the Euclidian distance.

### 2.6. Second Study Phase

For the qualitative analysis, two focus groups will be formed in each hospital participating in the study. One group will be formed by patients receiving specialist care and the other group will be formed by patients receiving only primary care. Therefore, the real sampling size will be approximately 140 people. We need to bear in mind that the principle of saturation will be used, that is to say, data collection will stop when it ceases to provide significant new information [31].

The sample will be selected intentionally, taking into account the criteria that allow for the collection of the different perceptions and expectations about the care received by the patients during the survivorship stage. The focus groups will be formed by an external coordinator and an external observer (members of the research team consisting of 6 to 8 patients per focus group).

Patients will be selected following the aforementioned specifications and will be summoned for the appointment, where first they will be informed by the researcher. They will also be required to sign an informed consent form, as patients who did not participate in the first phase may participate in this one. The data will be kept by the researchers in each hospital, as the relevant data protection law states.

The group coordinator, by using a structured script agreed upon by the whole research team for the different focus groups, will conduct the meeting, taking account of the information saturation as a criterion for, first, changing the topic, and last, ending the meeting. The script will be structured, keeping in mind the physical, emotional, work, economic, existential, and social needs, and the care received. The script will also give the patient the opportunity to provide information not collected or considered during the previous stages. During this phase, data regarding the satisfaction, clinical safety, and care quality perceived by the patient will be collected. We will register the data in digital records for each focus group.

This second study phase will be carried out for 3 months, from September 2024 to November 2024.

#### Data Analysis of the Second Study Phase

Inductive analysis based on grounded theory, known as the constant comparative method, will be used. This method consists of data collection based on a coding process, coding (meaning the process through which the information collected during the research is analysed and clustered in categories) and analysis in a systematic way, contrasting incidents, categories, hypotheses, and properties that arise during the collection and analysis process. The analysis method is based on a coding process that the main researcher will conduct together with another researcher, and it is divided into three phases: open, axial, and selective.

- Open coding: data will be divided and coded into concepts and categories will be developed. During the analysis process, new data will be compared to previously coded data in order to control the uniformity of the collection.
- Axial coding: we will go from intercomparing data to comparing data with categories resulting from previous comparisons. When categories are related together, hypotheses are created. If the linkages obtained are insufficient, theoretical sampling will continue in the search for new cases that provide more information and allow for explaining concepts and specifying the theory. To achieve the interaction between what is known and what needs to be known, the collection and analysis of data will be conducted simultaneously until reaching theoretical saturation.
- Selective coding: categories will be integrated to reduce the number of concepts and to identify the central and main categories.

### 2.7. Limitations

The main limitations of the study arise from the cross-sectional descriptive design used, and the questionnaire used as an instrument, which is subject to be biased due to deference, central tendency, social desirability, and logical error. To minimize these risks in the information prior to the completion of the questionnaire, patients shall be reminded that their answers will be confidential, that there are no right or wrong answers, and their doubts will be clarified.

The aim of the joint design of the study is, among others, to provide a methodological triangulation in order to minimize and control potential bias.

### 2.8. Ethical and Legal Aspects

This study will thoroughly comply with all ethical and legal requirements in Spain: Act 41/2002 regarding the Autonomy of the Patient, Organic Act 3/2018 of 5 December regarding Personal Data Protection and Guarantee of Digital Rights, and Regulation 2016/679 of the EU Parliament and Council, of 27 April 2016, regarding Protection of Natural Persons with respect to the treatment of personal data and the free circulation of such data, as well as the rest of the relevant and applicable regulations.

This study will be developed in accordance with the protocol and good clinical practice standards regarding human research, as stated in the Declaration of Helsinki.

The involvement of the patients is completely voluntary. They will be informed beforehand, and they may abandon the study at any time with no explanation, which will not affect their medical assistance.

The data obtained during the research will be processed exclusively by members of the research team, and any information that may identify any of the patients will be dissociated.

All patients will receive information on the project, their degree of implication, and the legal requirements in a document included with the informed consent form, which they must sign if they are willing to participate in the study.

All of the data will be handled following the Organic Act 3/2018 of 5 December regarding Personal Data Protection and Guarantee of Digital Rights, with data anonymization and preservation in separated files, that will be saved and guarded in independent and secure devices.

## 3. Discussion

This study will provide a better knowledge of the quality of life of these patients, as well as their level of unmet and even unexpressed needs, in order to develop effective strategies and interventions that allow for the implementation of adapted care plans that include such unexpressed needs. This study will also allow for the creation and development of assessment methods for health results from the patient’s perspective and experience.

When the CESC questionnaire results were assessed, around 80% of patients stated at least one unmet need during the post-treatment stage, in accordance with other studies [29,32] that reported this issue affecting close to two-thirds of the patients.

On the other hand, all resources provided by both health professionals and institutions are available. However, to achieve the proposed goals as soon as possible, we are seeking research funds.

## 4. Conclusions

Obtaining this sort of information may allow us to make a precise map of the health needs perceived by long-term cancer survivors, to spot undetected and therefore unmet needs, to acknowledge their expectations of the health care received during their survivorship stage, and to have information to improve their care quality.

## Figures and Tables

**Table 1:** Hospitals participating in the study.

Hospital	City
Hospital Regional Universitario de Malaga	Malaga
Hospital Clínico Virgen de la Victoria	Malaga
Hospital Consorcio Sanitario del Maresme	Barcelona
Hospital Universitario de Navarra	Pamplona
Hospital San Carlos	Madrid
Hospital Universitario de Donostia	San Sebastián
Hospital Torrecárdenas	Almería
Hospital Juan Ramón Jiménez	Huelva
Hospital Universitario de Orense	Orense
Hospital Marqués de Valdecilla	Santander

Source: self-made.

**Table 2:** Inclusion and exclusion criteria.

Inclusion Criteria	Exclusion Criteria
Having suffered from cancer for a period longer than 5 years.	Relapse of the same disease or metastasis that requires a new treatment.
Being 18 years of age or older.	Problems understanding the Spanish language.
Having completed the relevant cancer treatments (chemotherapy, radiation therapy, …) 2 or more years prior to the study.	Psychological and mental problems that impede the filling of questionnaires.
	Second malignancy.
Being disease-free at the moment of data collection.	Death before or during the study.

Source: self-made.

**Table 3:** Variables.

	Variable	Nature	Variable Expression
Socio-demographic variables	Belongs to patients’ associations	Quantitative dichotomous	Yes/no
	Volunteering	Quantitative dichotomous	Yes/no
	Gender	Quantitative dichotomous	Male/female
	Marital status	Quantitative polytomous	Single/Married/Widow-widower/Separated/Divorced/ Partnered
	Education	Quantitative polytomous	Primary Ed./Secondary Ed./Higher-University Ed./No answer
	Employment	Quantitative polytomous	Self-employed or working for others/Unemployed/Retired/Disability to work/Student/Other
	Rank of age	Quantitative dichotomous	Less or more than 40 years
Clinical variables	Income	Quantitative polytomous	Under 10,000/12,001–24,000/24,001–36,000/Above 36,000/No answer
	Time from diagnosis to completion of treatment	Quantitative continuous	Months
	Time from completion of treatment	Quantitative continuous	Years
	Type of cancer	Quantitative polytomous	Type of cancer
	Type of treatment	Quantitative polytomous	Chemotherapy—Radiation therapy—Surgery—Bone marrow transplant—Other
	Co-morbidity	Quantitative polytomous	Obesity—Hypertension—Bone problems—Diabetes—Cardiovascular diseases
	Patient survivorship	Quantitative dichotomous	>5 years, >10 years

Source: self-made.

## Data Availability

This manuscript was previously posted to Research Square: doi: https://doi.org/10.21203/rs.3.rs-2868006/v1 (accessed on 20 May 2023).
